# Incidence of urinary retention during treatment with single tablet combinations of solifenacin+tamsulosin OCAS™ for up to 1 year in adult men with both storage and voiding LUTS: A subanalysis of the NEPTUNE/NEPTUNE II randomized controlled studies

**DOI:** 10.1371/journal.pone.0170726

**Published:** 2017-02-06

**Authors:** Marcus J. Drake, Matthias Oelke, Robert Snijder, Monique Klaver, Klaudia Traudtner, Karin van Charldorp, Dominique Bongaerts, Philip Van Kerrebroeck

**Affiliations:** 1 University of Bristol, Bristol, United Kingdom; 2 Bristol Urological Institute, Bristol, United Kingdom; 3 Hannover Medical School, Hannover, Germany; 4 Astellas Pharma Europe B.V., Leiden, The Netherlands; 5 Maastricht University Medical Center, Maastricht, The Netherlands; Eberhard Karls University, GERMANY

## Abstract

**Introduction:**

The emergence of urinary retention (UR), specifically acute urinary retention (AUR), has been a concern when treating men with lower urinary tract symptoms (LUTS) with antimuscarinic drugs.

**Materials and methods:**

In NEPTUNE (12-week, double-blind), men (≥45 years) with LUTS were randomized to receive tamsulosin oral-controlled absorption system (TOCAS) 0.4 mg, fixed-dose combination (FDC) of solifenacin (Soli) 6 mg + TOCAS 0.4 mg, FDC Soli 9 mg + TOCAS 0.4 mg, or placebo. In NEPTUNE II (40-week, open-label extension of NEPTUNE), continuing patients received 4-week FDC Soli 6 mg + TOCAS, then FDC Soli 6 mg or 9 mg + TOCAS for the remainder of the study, switchable every 3 months.

**Results:**

Across both studies, 1208 men received ≥1 dose of FDC Soli 6 mg or 9 mg + TOCAS for up to 52 weeks; 1199 men completed NEPTUNE and 1066 received ≥1 dose in NEPTUNE II. In total, 13 men (1.1%; 95% CI, 0.6%–1.8%) reported a UR event while receiving FDC, eight of which were AUR (0.7%; 95% CI, 0.3%–1.3%, incidence 7/1000 man-years). Six men reported UR events while taking Soli 6 mg + TOCAS (three AUR), and seven men reported a UR event while taking Soli 9 mg + TOCAS (five AUR). One man developed AUR while taking TOCAS alone and four reported UR (three AUR) during placebo run-in. Most AUR/UR events occurred within 4 months of treatment initiation.

**Conclusions:**

FDC Soli and TOCAS was associated with a low rate of UR and AUR in men with LUTS.

## Introduction

‘Lower urinary tract symptoms’ (LUTS) is an umbrella term that encompasses a broad spectrum of urinary symptoms, including storage symptoms (e.g., urgency, increased daytime frequency, nocturia), voiding symptoms (e.g., slow stream, hesitancy, intermittency, terminal dribble), and post-micturition symptoms (feeling of incomplete bladder emptying and post-micturition dribbling) [1]. Voiding symptoms can be effectively treated with α_1_-adrenoceptor antagonists (α_1_-blockers), whereas storage symptoms respond well to muscarinic receptor antagonists (antimuscarinics) [2, 3]. However, many patients have both storage and voiding LUTS and may be undertreated for the storage component [4] because of a perceived increased risk of acute urinary retention (AUR), defined as ‘a painful, palpable, or percussible bladder when the patient is unable to pass any urine’ [1], with antimuscarinics [5].

Reported AUR rates in clinical trials with antimuscarinics and α_1_-blockers in men with LUTS associated with benign prostatic hyperplasia (LUTS/BPH) vary between studies and range from 0% to 1.9%, corresponding to an incidence rate of 0–78.2/1000 man-years [6]. The majority of trials evaluating antimuscarinics and α_1_-blocker combination therapy in men with LUTS/BPH are of short duration (≤12 weeks). The 12-week NEPTUNE [7] and 40-week NEPTUNE II [8] studies assessed men with storage and voiding LUTS/BPH treated with two fixed-dose combinations (FDCs) of the antimuscarinic solifenacin (Soli) and an oral-controlled absorption system formulation of the α_1_-blocker tamsulosin (TOCAS). To our knowledge, these are the only clinical trials that have studied the combination of an antimuscarinic and an α_1_-blocker for a period of up to 52 weeks. Thereby, the studies provide important new information regarding the long-term efficacy and tolerability of combined use of antimuscarinics and α_1_-blockers in men with LUTS. The aim of the current analysis was to evaluate the rate and time of onset of UR and the incidence of AUR cases (defined as those cases of UR that required catheterization) occurring over a period of up to 1 year. Furthermore, we assessed the relationship between the development of AUR and several baseline characteristics of the NEPTUNE study population that are known risk factors.

## Patients and methods

Patient characteristics and study results for the NEPTUNE studies have been published previously [7]. In brief, men aged ≥45 years were eligible to enter the studies if they had a diagnosis of LUTS/BPH for at least 3 months, a total International Prostate Symptom Score (IPSS) ≥13, ≥2 urgency episodes/24 h with Patient Perception of Intensity of Urgency Scale (PPIUS) grade 3 or 4 (i.e., severe urgency or urgency incontinence), and ≥8 micturitions/24 h, as well as an ultrasound-estimated prostate volume <75 mL, a maximum urinary flow rate (Q_max_) of 4.0–12.0 mL/s, and a post-void residual (PVR) volume of ≤150 mL.

**Fig 1:**
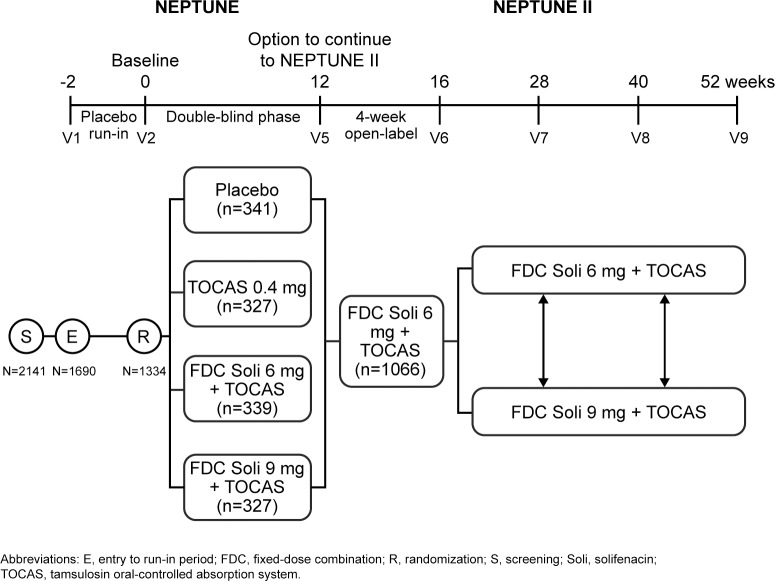
Study design of NEPTUNE and NEPTUNE II.

The NEPTUNE study (ClinicalTrials.gov identifier: NCT01018511, https://clinicaltrials.gov/ct2/show/NCT01018511?term=NCT01018511&rank=1) was a 12-week, randomized, double-blind, placebo-controlled, phase 3 trial that investigated the efficacy and safety of a once-daily FDC of Soli 6 mg or 9 mg and TOCAS 0.4 mg [7]. Patients were randomized 1:1:1:1 using a computer-generated randomized list to receive TOCAS 0.4 mg monotherapy, FDC Soli 6 mg + TOCAS 0.4 mg, FDC Soli 9 mg + TOCAS 0.4 mg, or placebo for 12 weeks. Patients completing NEPTUNE had the option to continue into the 40-week, open-label NEPTUNE II extension study (ClinicalTrials.gov identifier: NCT01021332, https://clinicaltrials.gov/ct2/show/NCT01021332?term=NCT01021332&rank=1) in which patients received FDC Soli 6 mg + TOCAS 0.4 mg for 4 weeks and could then choose to remain on Soli 6 mg + TOCAS 0.4 mg or increase their dose to Soli 9 mg + TOCAS 0.4 mg [8]. Patients were permitted to switch between doses at each subsequent 3-monthly visit (**Fig 1**). Data on FDC treatment from the NEPTUNE and NEPTUNE II studies have been combined to cover the period of potential drug exposure of up to 52 weeks. The NEPTUNE studies were approved by independent ethics committees and were conducted in accordance with the Declaration of Helsinki; written informed consent was obtained from all patients.

The safety analysis set (SAF) included all patients who received ≥1 dose of FDC in NEPTUNE and/or NEPTUNE II and who had any safety data reported after the first dose. Adverse events (AEs) were coded using MedDRA (Version 12.1) preferred terms. All cases of AUR, a subgroup of UR cases that required catheterization, were reported as serious AEs. No specific definitions for UR were provided. Analyses were performed according to exposure to FDC (up to 52 weeks).

The occurrence of AUR/UR during the 1-year treatment period is illustrated in cumulative incidence plots. A Cox-regression model was used to evaluate whether specific patient and baseline disease characteristics (i.e., prostate-specific antigen [PSA], prostate volume, Q_max_, total IPSS, PVR, bladder voiding efficiency [calculated as voided volume/(voided volume + PVR)], age) were associated with an increase in the risk of developing AUR/UR; Pearson correlation coefficients were derived between all characteristics of interest to understand the relationship between the potential risk factors. All 95% confidence intervals for proportions were calculated using the exact method.

## Results

### Patient disposition

Of 1334 men randomized into NEPTUNE, 1328 received ≥1 dose of study medication and were included in the SAF, and 1199 completed the 12-week, double-blind study. A total of 1066 patients received ≥1 dose of open-label FDC therapy. Of these, 960 patients (90.1%) completed the 40-week extension study and 106 patients (9.9%) discontinued, mainly due to AEs (n = 43, 4.0%), withdrawal of consent (n = 23, 2.2%), or lack of efficacy (n = 19, 1.8%). In total, 1208 patients received ≥1 dose of FDC therapy during NEPTUNE and/or NEPTUNE II [8].

### Urinary retention rates in NEPTUNE and NEPTUNE II

During the placebo run-in period, four men experienced UR, three cases of which were AUR and led to discontinuation from the study. Across both NEPTUNE and NEPTUNE II, 14 men experienced episodes of UR: 13 of the 1208 men (1.1%; 95% CI, 0.6%–1.8%) who received FDC Soli 6 mg or 9 mg + TOCAS (**Table 1**) and one man who received TOCAS monotherapy. For 10 of the 13 patients experiencing UR while on FDC, the investigator judged the events to be possibly or probably related to study medication. Of these 13 men, eight (0.7%; 95% CI, 0.3%–1.3%; incidence 7/1000 man-years) developed AUR, four cases of which required hospitalization. Detailed information about each individual who experienced UR or AUR can be found in **Table 2**.

**Table 1:** Numbers of patients on FDC Soli + TOCAS treatment with urinary retention or AUR in NEPTUNE and NEPTUNE II.

	**Treatment arm**		**Total**
	**FDC Soli 6 mg + TOCAS**	**FDC Soli 9 mg + TOCAS**	
NEPTUNE (12 weeks)	(n = 337)	(n = 324)	(n = 661)
UR, n (%, [95% CI])	3 (0.9, [0.2–2.6])	4 (1.2, [0.3–3.1])	7 (1.1, [0.4–2.2])
AUR^a^, n (%, [95% CI])	1 (0.3, [0.0–1.6])^b^	3 (0.9, [0.2–2.7])^c^	4 (0.6, [0.2–1.5])^d^
	**Dose at onset of adverse event**		**Total**
	**FDC Soli 6 mg +TOCAS**^**e**^	**FDC Soli 9 mg +TOCAS**^**e**^	
NEPTUNE II (40 weeks)	(n = 1066)		(n = 1066)
UR, n (%, [95% CI])	3	3	6 (0.6, [0.2–1.2])
AUR^a^, n (%, [95% CI])	2	2	4 (0.4, [0.1–1.0])
	**Dose at onset of adverse event**		**Total**
	**FDC Soli 6 mg +TOCAS**^**e**^	**FDC Soli 9 mg +TOCAS**^**e**^	
Total (up to 52 weeks)	(n = 1208)		(n = 1208)
UR, n (%, [95% CI])	6	7	13 (1.1, [0.6–1.8])
AUR^a^, n (%, [95% CI])	3	5	8 (0.7, [0.3–1.3])^f^

95% CIs were calculated using the exact method.

^a^ AUR cases were a subgroup of UR cases that required catheterization.

^b^ Incidence of 13/1000 man-years (95% CI, 6.9–22.2). Previously reported incidence rate of 0 man-years; difference is accounted for by the inclusion of an AUR case deemed not related to study medication that was not included in the previous analysis [6].

^c^ Incidence of 40/1000 man-years (95% CI, 27.7–53.3).

^d^ Incidence of 26/1000 man-years (95% CI, 17.0–38.1). Previously reported incidence rate of 19 man-years; difference is accounted for by the inclusion of an AUR case deemed not related to study medication that was not included in the previous analysis [6].

^e^ Percentages and incidence per 1000 man-years cannot be calculated for the individual doses as patients did not use either FDC 6 mg or 9 mg for a whole year; furthermore, patients selected their dose (i.e., were not randomized), so the two dose groups cannot be assumed to be comparable.

^f^ Incidence of 7/1000 man-years (95% CI, 2.8–14.4).

Note: One additional patient on TOCAS experienced AUR during the 12-week double-blind period (0.3%; incidence of 13/1000 man-years). Abbreviations: AUR, acute urinary retention; CI, confidence interval; FDC, fixed-dose combination; Soli, solifenacin; TOCAS, tamsulosin oral-controlled absorption system; UR, urinary retention.

**Table 2:** Summary of patients on FDC Soli + TOCAS treatment with UR/AUR in NEPTUNE and NEPTUNE II, listed in chronological order of appearance.

Days on FDC at onset of AE	FDC dose at onset of AE (mg)	AUR/UR^a^	Discontinued study	Patient age (years)	Total IPSS at baseline	PVR volume at baseline (mL)	Q_max_ at baseline (mL/s)	Prostate volume at baseline (mL)	PSA (ng/mL)
**NEPTUNE (double-blind FDC Soli + TOCAS)**									
6	9	AUR	No	72	14	28	9.4	74	2.8
12	9	AUR	No	67	21	50	9.8	54	1.1
24	9	AUR	Yes						
22	6	AUR	No	62	20	148	7.0	48	5.7
31	6	UR	Yes	54	24	122	5.2	15	0.7
39	9	AUR	Yes	75	16	15	7.8	35	4.3
53	9	UR	No	68	17	134	10.6	24	2.2
77	6	UR	No	66	22	75	7.9	46	1.4
**NEPTUNE II (open-label FDC Soli + TOCAS)**									
106	6^b^	AUR	Yes	79	15	33	6.3	33	4.5
117	6^c^	UR	Yes	67	29	74	6.4	18	2.4
191^d^	9^e^	UR	No	58	18	48	7.0	65	5.2
194	6^e^	AUR	Yes	63	15	52	9.0	55	4.9
254^f^^,^^g^	9^e^	AUR	No	67	27	50	7.6	71	5.3
347^h^	9^b^	AUR	Yes	64	18	103	9.5	56	10.5

^a^ AUR cases were a subgroup of UR cases that required catheterization.

^b^ Treatment received in the NEPTUNE trial was FDC Soli 6 mg + TOCAS.

^c^ Treatment received in the NEPTUNE trial was placebo.

^d^ Patient increased to FDC Soli 9 mg + TOCAS for 67 days prior to onset.

^e^ Treatment received in the NEPTUNE trial was TOCAS.

^f^ Patient increased to FDC Soli 9 mg + TOCAS for 141 days prior to onset.

^g^ High alcohol consumption noted the evening before UR onset.

^h^ Patient increased to FDC Soli 9 mg + TOCAS for 149 days prior to onset.

Note: One additional patient on TOCAS experienced AUR during the 12-week double-blind period. The patient was hospitalized for osteoarthritis and later hip arthroplasty; 2 days after hip arthroplasty, he developed UR. The patient did not discontinue the trial. Age: 64 years; total IPSS at baseline: 30; PVR volume: 87 mL; Qmax: 6.2 mL/s; baseline prostate volume: 25 mL; PSA concentration: 0.3 ng/mL.

Abbreviations: AE, adverse event; AUR, acute urinary retention; FDC, fixed-dose combination; IPSS, International Prostate Symptom Score; PSA, prostate-specific antigen; PVR, post-void residual; Qmax, maximum urinary flow rate; TOCAS, tamsulosin oral-controlled absorption system; UR, urinary retention.

Of the 14 men experiencing UR, eight experienced episodes during the 12-week, double-blind NEPTUNE study (**Table 1**). Three men (0.9%) receiving FDC Soli 6 mg + TOCAS (one AUR, 0.3%, corresponding to an incidence rate of 13/1000 man-years), four men (1.2%) receiving FDC Soli 9 mg + TOCAS (three AUR, 0.9%, corresponding to an incidence rate of 40/1000 man-years), and one man (0.3%, corresponding to an incidence rate of 13/1000 man-years) of the 326 men receiving TOCAS monotherapy experienced an event of AUR.

Additionally, six men experienced UR, four of which were AUR, during the 40-week, open-label NEPTUNE II study. Three men experienced the event onset of UR (two of which were AUR) while receiving FDC Soli 6 mg + TOCAS. Similarly, three men experienced the event onset of UR (two of which were AUR) while receiving FDC Soli 9 mg + TOCAS (**Table 1**). Two men who experienced UR in NEPTUNE continued into the NEPTUNE II extension study and did not experience any further episodes. None of the men experiencing UR in NEPTUNE II had prior episodes in the 12-week double-blind NEPTUNE study.

### FDC dose duration at onset of urinary retention

**Fig 2:**
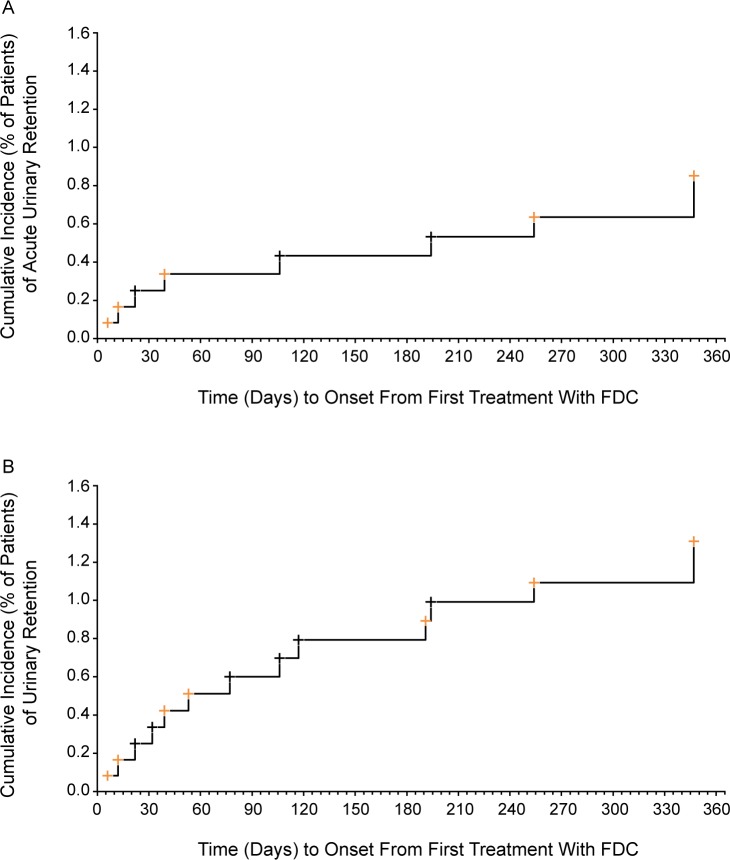
**Cumulative Incidence of AUR (A) and UR (B) with FDC Soli 6 mg or 9 mg + TOCAS.** (A) Acute urinary retention. (B) Urinary retention. Black markers indicate 6 mg FDC dose at time of AUR/UR onset; orange markers indicate 9 mg FDC dose at time of AUR/UR onset. AUR cases were a subgroup of UR cases that required catheterization. Abbreviation: FDC, fixed-dose combination.

The duration of FDC exposure at the time of onset of UR varied considerably (median 77 days, range 6–347 days); however, the majority of patients developed UR within the first 4 months of treatment (9 of 13, 69.2%). Four men (30.8%) had UR after at least 4 months of treatment, one on Soli 6 mg + TOCAS and three on Soli 9 mg + TOCAS. Cumulative incidence plots for AUR and UR (**Fig 2A and 2B**) were developed to illustrate the timing of their occurrence in relation to the duration of treatment with FDC Soli 6 mg or 9 mg + TOCAS.

### Baseline risk factors for acute urinary retention

The mean baseline values for potential risk factors for AUR of patients with UR or AUR on FDC Soli + TOCAS (NEPTUNE/NEPTUNE II) and of the total NEPTUNE SAF population are presented in **S1 Table**. As some of these risk factors were found to be highly correlated with each other (**S2 Table**), a post-hoc Cox-regression model, assessing risk of developing UR or AUR, was derived separately for each potential risk factor (**Table 3**). The results of the Cox-regression analysis indicated that decreased bladder voiding efficiency (BVE) and increased PSA concentration, PVR volume, and prostate volume at baseline were associated with an increased risk of developing AUR and UR; other risk factors tested (i.e., Q_max_, total IPSS, age) did not show evidence for an increased risk of AUR or UR.

**Table 3:** Risk of developing AUR/UR by potential risk factors.

	AUR*			UR^‡^		
	Hazard Ratio	95% CI	*P*-value	Hazard Ratio	95% CI	*P*-value
**Age, years**	1.05	0.94–1.16	0.40	1.02	0.94–1.12	0.62
**Prostate volume, mL**	1.08	1.02–1.13	**0.004**	1.04	1.00–1.08	**0.048**
**Q**_**max**_**, mL/s**	0.85	0.60–1.21	0.36	0.78	0.59–1.03	0.08
**Total IPSS**	0.97	0.80–1.17	0.74	1.05	0.91–1.20	0.50
**PSA concentration, ng/mL**	1.41	1.16–1.71	**<0.001**	1.37	1.15–1.63	**<0.001**
**PVR volume, mL**	1.02	1.00–1.03	0.06	1.02	1.01–1.04	**<0.001**
**BVE, %**	0.94	0.90–0.99	**0.03**	0.93	0.89–0.97	**<0.001**

*Total subjects (N = 1202), censored (n = 1194)

^‡^Total subjects (N = 1202), censored (n = 1189).

Abbreviations: BVE, bladder voiding efficiency; CI, confidence interval; IPSS, International Prostate Symptom Score; PSA, prostate-specific antigen; PVR, post-void residual; Q_max_, maximum urinary flow rate.

Bold indicates statistical significance (*P*<0.05).

Although an association between prostate volume and increased AUR/UR was determined, the overall rates of UR and AUR were low in all patients regardless of prostate volume. In the 12-week double-blind study, the AUR rates on the FDC of Soli 6 mg + TOCAS were 0% (0/203) and 0.7% (1/134) for patients with a prostate volume of ≤40 mL and >40 mL, respectively. The AUR rates on the FDC of Soli 9 mg + TOCAS were 0.5% (1/185) and 1.4% (2/138) for patients with a prostate volume of ≤40 mL and >40 mL, respectively.

## Discussion

Traditionally, the use of antimuscarinics in men with LUTS/BPH, benign prostatic enlargement, and/or benign prostatic obstruction has been limited owing to fears of AUR [3]. This belief, however, has been questioned by a number of studies performed over the past decade demonstrating promising efficacy and safety of combination therapy using antimuscarinics and α_1_-blockers [5]. The vast majority of data are from short-term studies (≤12 weeks), even though treatment with antimuscarinics and α_1_-blockers combination therapy is a long-term option. NEPTUNE II, together with NEPTUNE, is the only long-term study to assess the efficacy and safety of 52 weeks of treatment with antimuscarinic and α_1_-blocker combination therapy. Patients with storage and voiding LUTS, Q_max_ <12 mL/s, PVR volume ≤150 mL, and prostate volume <75 mL were eligible for inclusion. We consider that these patients are typical candidates for combination therapy in routine clinical practice.

In NEPTUNE and NEPTUNE II, treatment with FDC Soli + TOCAS therapy for up to 52 weeks was associated with low rates of UR (1.1%) and AUR (0.7%, corresponding to an incidence of 7/1000 man-years). Most cases occurred within the first 4 months after treatment initiation, and only one patient experienced UR after this period with the now-approved FDC dosage (Soli 6 mg + TOCAS 0.4 mg). These results are in agreement with those of The Health Improvement Network (THIN) database analysis, which reported the incidence of AUR in men treated with oral antimuscarinics. This analysis found that the relative risk of AUR was highest in early treatment (first 30 days) and lowered with time [9]. Consequently, patients at risk for UR should be initially treated with caution and counselled for the symptoms and signs of UR [10]. The long-term incidence of AUR on the FDC Soli 6 mg + TOCAS dose cannot be calculated owing to the permitted dose changes during the 40-week open-label period; however, the total incidence in the patient population studied was low (7/1000 man-years) despite the inclusion of cases reported while on the higher Soli 9 mg + TOCAS dose.

Rates of UR and AUR were similarly low during the first 12 weeks of the double-blind NEPTUNE study. Events of UR were reported in only 7 of 661 patients receiving FDC: three events (0.9%) occurred with FDC 6 mg and four events (1.2%) occurred with FDC 9 mg. Four of these UR events were AUR: one (0.3%, incidence 13/1000 man-years) occurred with FDC 6 mg and three (0.9%, incidence 40/1000 man-years) occurred with FDC 9 mg. These data are consistent with previously published AUR rates and incidences for treatment with Soli + TOCAS [6, 11–13]. The AUR rates reported here are also consistent with other 12-week studies evaluating tolterodine (another antimuscarinic) in combination with tamsulosin in men with LUTS (0–0.9%) [14–16]. The AUR incidence in the NEPTUNE studies, regardless of FDC dose, is also in the same range as the incidence of AUR reported for the general, symptomatic, but untreated male LUTS population (18.3–35.9/1000 man-years) [6]. However, it should be noted that inclusion and exclusion criteria varied between different clinical trials, and thus populations are likely to have different propensities for UR.

Rates of UR and AUR in the NEPTUNE and NEPTUNE II studies may have been affected by the inclusion and exclusion criteria: for example, patients with a PVR >150 mL were excluded from the study, potentially lowering the overall risk of retention in this population. In most other studies with antimuscarinics, baseline PVR volume was also restricted, typically to ≤200 mL. Thus, additional research would be required to comment on the use of combination therapy in men with higher baseline PVR volumes. Despite the exclusion of patients with a prostate volume ≥75 mL at baseline, mean/median prostate volume at baseline was 38.1/36.0 mL (range: 9–74 mL), which is comparable with patients investigated in the Medical Therapy of Prostatic Symptoms (MTOPS) study (mean prostate volume of 36.3 mL [17]), indicating that prostate volume was clearly increased for the majority of patients in this study, although not typical for patients with LUTS/BPH.

A number of known risk factors for UR, as described in the MTOPS and Olmsted County studies [17, 18], were present in the total NEPTUNE population at baseline, suggesting a propensity for UR in this population. While age and total IPSS were not shown to be associated with risk for AUR, decreased BVE, and increased PSA, PVR, and prostate volume at baseline were significantly associated with increasing risk of developing AUR/UR by cox-regression analysis. Although more men with prostate volume >40 mL experienced AUR than men whose baseline prostate volume was ≤40 mL, the overall rate of AUR across the entire study was low. As the low rates of AUR may impact the ability to accurately assess risk factors, the results of this post-hoc analysis should therefore be interpreted with caution.

Following this clinical trial program, the FDC tablet of Soli 6 mg + TOCAS 0.4 mg has been registered in 42 countries (Vesomni™ or Urizia™), in most cases for the treatment of moderate to severe storage (urgency, increased micturition frequency) and voiding LUTS associated with BPH in men who are not adequately responding to treatment with monotherapy. Registration procedures in other countries are ongoing.

## Conclusions

Results from the NEPTUNE and NEPTUNE II studies suggest that treatment for up to 52 weeks with an FDC tablet containing Soli 6 mg or 9 mg with TOCAS 0.4 mg was well tolerated and associated with low rates of UR and AUR in men with LUTS. The overall incidence of UR was low, occurring in only 13 of 1208 patients (1.1%) receiving FDC Soli + TOCAS in either NEPTUNE or NEPTUNE II, with eight of those patients (0.7%, incidence 7/1000 man-years) requiring catheterization (AUR). Decreased BVE and increased PSA, PVR, and prostate volume at baseline were associated with an increased risk of developing AUR/UR. However, it should be noted that the low rates of AUR/UR observed in this study limit the accuracy of risk assessment. Most cases occurred within 4 months of treatment initiation, suggesting no increase in the risk of AUR with long-term treatment compared with 12 weeks of treatment.

## Supporting information

S1 Table Mean baseline characteristics of patients with AUR/UR on FDC Soli + TOCAS (NEPTUNE/NEPTUNE II) and the total NEPTUNE safety analysis set population. (DOCX)

S2 Table Pearson correlation matrix for potential risk factors. (DOCX)

## References

[1] Abrams P, Cardozo L, Fall M, Griffiths D, Rosier P, Ulmsten U, et al The standardisation of terminology of lower urinary tract function: report from the Standardisation Sub-committee of the International Continence Society. Neurourol Urodyn. 2002;21(2):167–78. 11857671 10.1002/nau.10052

[2] Drake MJ. Male lower urinary tract symptoms associated with overactive bladder. Can Urol Assoc J. 2012;6(5 Suppl 2):S136–7. 23092778 10.5489/cuaj.12202 PMC3481948

[3] Oelke M, Bachmann A, Descazeaud A, Emberton M, Gravas S, Michel MC, et al EAU guidelines on the treatment and follow-up of non-neurogenic male lower urinary tract symptoms including benign prostatic obstruction. Eur Urol. 2013;64(1):118–40. 10.1016/j.eururo.2013.03.004 23541338

[4] Helfand BT, Evans RM, McVary KT. A comparison of the frequencies of medical therapies for overactive bladder in men and women: analysis of more than 7.2 million aging patients. Eur Urol. 2010;57(4):586–91. 10.1016/j.eururo.2009.12.025 20036783

[5] Kaplan SA, Roehrborn CG, Abrams P, Chapple CR, Bavendam T, Guan Z. Antimuscarinics for treatment of storage lower urinary tract symptoms in men: a systematic review. Int J Clin Pract. 2011;65(4):487–507. 10.1111/j.1742-1241.2010.02611.x 21210910

[6] Oelke M, Speakman MJ, Desgrandchamps F, Mamoulakis C. Acute Urinary Retention Rates in the General Male Population and in Adult Men With Lower Urinary Tract Symptoms Participating in Pharmacotherapy Trials: A Literature Review. Urology. 2015;86(4):654–65. 10.1016/j.urology.2015.06.025 26142712

[7] van Kerrebroeck P, Chapple C, Drogendijk T, Klaver M, Sokol R, Speakman M, et al Combination therapy with solifenacin and tamsulosin oral controlled absorption system in a single tablet for lower urinary tract symptoms in men: efficacy and safety results from the randomised controlled NEPTUNE trial. Eur Urol. 2013;64(6):1003–12. 10.1016/j.eururo.2013.07.034 23932438

[8] Drake MJ, Chapple CR, Sokol R, Oelke M, Traudtner K, Klaver M, et al Long-term safety and efficacy of single tablet combinations of solifenacin and tamsulosin OCAS in men with storage and voiding LUTS: results from the NEPTUNE study and NEPTUNE II open label extension. Eur Urol. 2014;67(2):262–70. 10.1016/j.eururo.2014.07.013 25070148

[9] Martin-Merino E, Garcia-Rodriguez LA, Masso-Gonzalez EL, Roehrborn CG. Do oral antimuscarinic drugs carry an increased risk of acute urinary retention? J Urol. 2009;182(4):1442–8. 10.1016/j.juro.2009.06.051 19683302

[10] Oelke M, Burger M, Castro-Diaz D, Chartier-Kastler E, Jimenez Cidre MA, McNicholas T, et al Diagnosis and medical treatment of lower urinary tract symptoms in adult men: applying specialist guidelines in clinical practice. BJU Int. 2012;110(5):710–8. 10.1111/j.1464-410X.2011.10808.x 22145967

[11] Kaplan SA, McCammon K, Fincher R, Fakhoury A, He W. Safety and tolerability of solifenacin add-on therapy to alpha-blocker treated men with residual urgency and frequency. J Urol. 2009;182(6):2825–30. 10.1016/j.juro.2009.08.023 19837435

[12] Kaplan SA, He W, Koltun WD, Cummings J, Schneider T, Fakhoury A. Solifenacin Plus Tamsulosin Combination Treatment in Men With Lower Urinary Tract Symptoms and Bladder Outlet Obstruction: A Randomized Controlled Trial. Eur Urol. 2013;63(1):158–65. 10.1016/j.eururo.2012.07.003 22831853

[13] Van Kerrebroeck PEV, Haab F, Angulo JC, Vik V, Katona F, Garcia-Hernandez A, et al Efficacy and safety of solifenacin plus tamsulosin OCAS in men with voiding and storage LUTS: Results from a phase 2, dose-finding study (SATURN). Eur Urol. 2013;64(3):398–407. 10.1016/j.eururo.2013.03.031 23537687

[14] Athanasopoulos A, Gyftopoulos K, Giannitsas K, Fisfis J, Perimenis P, Barbalias G. Combination treatment with an alpha-blocker plus an anticholinergic for bladder outlet obstruction: a prospective, randomized, controlled study. J Urol. 2003;169(6):2253–6. 10.1097/01.ju.0000067541.73285.eb 12771763

[15] Chapple CR, Herschorn S, Abrams P, Sun F, Brodsky M, Guan Z. Tolterodine treatment improves storage symptoms suggestive of overactive bladder in men treated with alpha-blockers. Eur Urol. 2009;56(3):534–41. 10.1016/j.eururo.2008.11.026 19070418

[16] Roehrborn CG, Kaplan SA, Jones JS, Wang JT, Bavendam T, Guan Z. Tolterodine extended release with or without tamsulosin in men with lower urinary tract symptoms including overactive bladder symptoms: effects of prostate size. Eur Urol. 2009;55(2):472–9. 10.1016/j.eururo.2008.06.032 18583022

[17] McConnell JD, Roehrborn CG, Bautista OM, Andriole GL Jr., Dixon CM, Kusek JW, et al The long-term effect of doxazosin, finasteride, and combination therapy on the clinical progression of benign prostatic hyperplasia. N Engl J Med. 2003;349(25):2387–98. 10.1056/NEJMoa030656 14681504

[18] Jacobsen SJ, Jacobson DJ, Girman CJ, Roberts RO, Rhodes T, Guess HA, et al Natural history of prostatism: risk factors for acute urinary retention. J Urol. 1997;158(2):481–7. 9224329 10.1016/s0022-5347(01)64508-7

