# Supplementary material for: Incidence of urinary retention during treatment with single tablet combinations of solifenacin+tamsulosin OCAS™ for up to 1 year in adult men with both storage and voiding LUTS: A subanalysis of the NEPTUNE/NEPTUNE II randomized controlled studies
Source: PLoS One. 2017 Feb 6;12(2):e0170726. doi: 10.1371/journal.pone.0170726 (PMC5293258; doi:10.1371/journal.pone.0170726)
Supplement: S1 Table — (DOCX) [file pone.0170726.s001.docx]

**S1 Table. Mean baseline characteristics of patients with AUR/UR on FDC Soli + TOCAS (NEPTUNE/NEPTUNE II) and the total NEPTUNE safety analysis set population**

|  | Total^a^ (N=1328) | UR on FDC (N=13) | AUR on FDC (N=8) |
| --- | --- | --- | --- |
| Age, years (range) | 65.4 (45–86) | 66.3 (54–79) | 68.6 (62–79) |
| Age ≤65, n (%) | 658 (49.5) | 5 (38.5) | 3 (37.5) |
| Age >65, n (%) | 670 (50.5) | 8 (61.5) | 5 (62.5) |
| Age ≥75, n (%) | 138 (10.4) | 2 (15.4) | 2 (25.0) |
| PVR volume, mL (range) | 36.5 (0–150) | 71.7 (15–148) | 59.9 (15–148) |
| Q_max_, mL/s (range) | 8.9 (4.0–19.2) | 8.0 (5.2–10.6) | 8.3 (6.3–9.8) |
| Prostate volume, mL (range) | 38.1 (9–74) | 45.7 (15–74) | 53.3 (33–74) |
| Total IPSS (SD) | 18.7 (4.5) | 19.7 (4.7) | 18.3 (4.3) |
| PSA concentration, ng/mL (range) | 2.3 (0.1–32.2) | 3.9 (0.7–10.5) | 4.9 (1.1–10.5) |

^a^ Total number of patients enrolled into NEPTUNE who were randomized and received at least one dose of study medication. Abbreviations: AUR, acute urinary retention; FDC, fixed-dose combination; IPSS, International Prostate Symptom Score; PSA, prostate-specific antigen; PVR, post-void residual; Q_max_, maximum urinary flow rate; SD, standard deviation, TOCAS, tamsulosin oral-controlled absorption system; UR, urinary retention.
