# Supplementary material for: Incidence of urinary retention during treatment with single tablet combinations of solifenacin+tamsulosin OCAS™ for up to 1 year in adult men with both storage and voiding LUTS: A subanalysis of the NEPTUNE/NEPTUNE II randomized controlled studies
Source: PLoS One. 2017 Feb 6;12(2):e0170726. doi: 10.1371/journal.pone.0170726 (PMC5293258; doi:10.1371/journal.pone.0170726)
Supplement: S2 Table — (DOCX) [file pone.0170726.s002.docx]

**S2 Table. Pearson correlation matrix for potential risk factors**

|  | **PVR volume (mL)** | **Prostate volume (mL)** | **Q_max_ (mL/s)** | **BVE** (%) | **PSA concentration (ng/mL)** | **Total IPSS** |
| --- | --- | --- | --- | --- | --- | --- |
| **PVR volume (mL)** | 1.000 |  |  |  |  |  |
| **Prostate volume (mL)** | 0.0814^a^ | 1.000 |  |  |  |  |
| **Q_max_ (mL/s)** | -0.1145^b^ | -0.0241 | 1.000 |  |  |  |
| **BVE (%)** | -0.9417^b^ | -0.0944^a^ | 0.1371^b^ | 1.000 |  |  |
| **PSA concentration (ng/mL)** | 0.0830^a^ | 0.3645^b^ | -0.0421 | -0.0928^a^ | 1.000 |  |
| **Total IPSS** | 0.1011^b^ | 0.0375 | -0.0470 | -0.1095^b^ | 0.0865^a^ | 1.000 |

Correlation values range between -1 and 1; a value of zero indicates no correlation.

^a^*P*<0.01; ^b^*P*<0.001

Abbreviations: BVE, bladder voiding efficiency; IPSS, International Prostate Symptom Score; PSA, prostate-specific antigen; PVR, post-void residual; Q_max_, maximum urinary flow rate.
